# Supplementary material for: HLA-DRB1*07:01 and *08:02 Alleles Confer a Protective Effect Against ACPA-Positive Rheumatoid Arthritis in a Latin American Admixed Population
Source: Biology (Basel). 2020 Dec 14;9(12):467. doi: 10.3390/biology9120467 (PMC7765073; doi:10.3390/biology9120467)
Supplement: Supplementary file 1 [file biology-09-00467-s001.pdf]

Table S1. Single-SNP association values base on the allele frequencies characterized for the RA patients vs. healthy controls.

SNP	Position	Gene. Location	A1	MAF RA	MAF controls	*p* value	P bonf	OR	L95	U95
rs4959028	32383138	BTNL2|HLA-DRA. Intergenic	G	0.06	0.08	0.13	1.00	0.73	0.49	1.10
rs2157335	32386131	BTNL2|HLA-DRA. Intergenic	A	0.04	0.06	0.16	1.00	0.71	0.44	1.15
rs9501626	32400344	BTNL2|HLA-DRA. Intergenic	A	0.15	0.19	0.02	0.49	0.73	0.55	0.96
rs3135335	32401845	BTNL2|HLA-DRA. Intergenic	G	0.26	0.34	$1.59x10^{-3}$	0.03	0.70	0.55	0.87
rs2027856	32402705	BTNL2|HLA-DRA. Intergenic	T	0.15	0.19	0.03	0.53	0.73	0.55	0.96
rs9268614	32402778	BTNL2|HLA-DRA. Intergenic	G	0.28	0.22	$5.54x10^{-3}$	0.11	1.39	1.10	1.76
rs3129867	32404220	BTNL2|HLA-DRA. Intergenic	G	0.25	0.33	$7.82x10^{-4}$	0.02	0.68	0.54	0.85
rs2395178	32405362	BTNL2|HLA-DRA. Intergenic	G	0.26	0.34	$1.59x10^{-3}$	0.03	0.70	0.55	0.87
rs3129882	32409530	HLA-DRA. Intron	G	0.38	0.46	$2.90x10^{-3}$	0.06	0.73	0.59	0.90
rs3129886	32410576	HLA-DRA. Intron	T	0.18	0.22	0.06	0.22	0.78	0.60	1.01
rs3129888	32411726	HLA-DRA. Intron	G	0.14	0.19	0.01	0.21	0.69	0.52	0.92
rs2239802	32411846	HLA-DRA. Intron	G	0.16	0.19	0.12	1.00	0.80	0.61	1.06
rs2395182	32413317	HLA-DRA|HLA-DRB1. Intergenic	G	0.16	0.19	0.12	1.00	0.80	0.61	1.06
rs9268844	32429153	HLA-DRA|HLA-DRB1. Intergenic	G	0.28	0.21	$2.40x10^{-3}$	0.05	1.44	1.14	1.83
rs3129768	32595083	HLA-DRB1|HLA-DQA1. Intergenic	G	0.13	0.18	0.01	1.00	0.69	0.52	0.92
rs7775228	32658079	HLA-DQB1|HLA-DQA2. Intergenic	C	0.12	0.16	$8.19x10^{-3}$	0.16	0.66	0.49	0.90
rs9275224	32659878	HLA-DQB1|HLA-DQA2. Intergenic	A	0.27	0.36	$2.31x10^{-4}$	$1.61x10^{-3}$	0.66	0.53	0.82
rs9275580	32679462	HLA-DQB1|HLA-DQA2. Intergenic	G	0.38	0.30	$1.16x10^{-3}$	0.02	1.43	1.15	1.77
rs2858332	32681161	HLA-DQB1|HLA-DQA2. Intergenic	G	0.29	0.33	0.12	1.00	0.84	0.67	1.05
rs3998158	32681992	HLA-DQB1|HLA-DQA2. Intergenic	C	0.34	0.26	$1.73x10^{-3}$	0.03	1.43	1.14	1.78

SNP = single nucleotide polymorphism; A1 = minor allele nucleotide; MAF = minor allele frequency; P BONF = *p* value using Bonferroni correction; OR = Odds ratio; L95 = lower bound on confidence interval for odds ratio; U95 = upper bound on confidence interval for odds ratio.

Publisher's Note: MDPI stays neutral with regard to jurisdictional claims in published maps and institutional affiliations.

© 2020 by the authors. Licensee MDPI, Basel, Switzerland. This article is an open access article distributed under the terms and conditions of the Creative Commons Attribution (CC BY) license (http://creativecommons.org/licenses/by/4.0/).
